# Differences in post-conflict behaviour in wolves and dog-admixed wolves

**DOI:** 10.1098/rsos.260767

**Published:** 2026-10-21

**Authors:** Federica Amici, Katja Liebal, Linda S. Oña, Arne Gretschzel, Tina Altdörfer, Mattia Iacuzzi, George Kamanga, Manon Delaunay, Paolo Ciucci

**Affiliations:** ^1^ Max-Planck-Institute for Evolutionary Anthropology, Leipzig, Saxony, Germany; ^2^ University of Leipzig Faculty of Life Sciences, Leipzig, Saxony, Germany; ^3^ Osnabrück University, Osnabrück, Niedersachsen, Germany; ^4^ Dipartimento di Biologia e Biotecnologie ‘Charles Darwin’, University of Rome La Sapienza, Rome, Lazio, Italy

**Keywords:** reconciliation, third-party affiliation, hybridization

## Abstract

Several species rely on mechanisms to resolve conflicts and reduce their negative consequences, including reconciliation and third-party affiliation (i.e. post-conflict affiliative interactions between victims and aggressors, and between victims and third parties, respectively). Here, we studied reconciliation and third-party affiliation in two groups of wolves (*n* = 20) and three groups of wolves introgressed with dogs (i.e. second- or later-generation backcrosses of wolf-dog hybrids to wolves; *n* = 13), in captivity. We conducted behavioural observations of post-conflict affiliative interactions using continuous focal sampling for 2 minutes following an aggression, and during matched-control periods. Our results showed that both admixed and non-admixed wolves engaged in post-conflict affiliation consistent with reconciliation and third-party affiliation. Although we found no evidence that admixed wolves showed lower reconciliation or third-party affiliation than non-admixed wolves, social factors had different modulatory effects in admixed and non-admixed wolves. In particular, stronger social bonds promoted post-conflict affiliation in non-admixed wolves, whereas greater rank distance was associated with higher post-conflict affiliation in admixed wolves. Moreover, post-conflict affiliation was more likely following high-intensity aggression in both admixed and non-admixed wolves, and after unidirectional conflicts in the latter. These findings indicate that dog introgression may influence post-conflict behaviour in wolves and suggest that, while both admixed and non-admixed wolves engage in post-conflict affiliation, the mechanisms underlying them may partially differ.

## 1. Introduction

In group-living animals, strong, enduring social relationships play a crucial role in enhancing individual fitness, positively affecting reproductive success, offspring survival and longevity (e.g. [1–5]). However, group-living also entails the risk of conflicts that may escalate into aggression, with potential negative consequences for the individuals involved, including the disruption of social relationships, increased risk of injury and elevated stress levels [6,7]. Therefore, group-living species may evolve mechanisms to resolve these conflicts, mitigate their negative consequences and ultimately contribute to maintaining group cohesion [6,7].

Post-conflict (PC) behaviour represents one of these mechanisms and refers to any behaviour occurring shortly after an aggressive interaction, involving former opponents (hereafter, victim and aggressor) or third parties. Post-conflict behaviour, in particular, includes reconciliation (i.e. affiliative interactions between victim and aggressor, shortly after a conflict) and third-party affiliation (i.e. affiliative interactions between victim and third parties, shortly after a conflict, which may be solicited or unsolicited by the victim [8]). Post-conflict behaviour may serve multiple functions. Reconciliation, for instance, may repair potential damage to social relationships caused by conflict, restore particularly valuable relationships and/or reduce stress arising from uncertainty in the relationship following conflict [9–13]. In turn, third-party affiliation may lower the likelihood of further aggression (towards the victim or in the group) and provide benefits to the victim and/or third parties by reducing their stress levels [14–16].

Several studies have investigated the occurrence of PC behaviour and have found evidence of both reconciliation and third-party affiliation across a wide range of taxa. Reconciliation was first documented in chimpanzees (*Pan troglodytes* [8]), and subsequently in several other primate species (e.g. [6,7,17,18]), as well as in domestic goats (*Capra hircus*; [19]), bottlenose dolphins (*Tursiops truncatus* [20]), spotted hyenas (*Crocuta Crocuta* [21,22]) and ravens (*Corvus corax* [23]). In canids, reconciliation has been shown in both wolves (*Canis lupus* [24–27]) and domestic dogs (*Canis lupus familiaris* [28,29]; but see [25]). Evidence of third-party affiliation is also widespread, although it is not always differentiated between solicited and unsolicited affiliation. So far, it has been reported in several species, including chimpanzees [14,30], other great ape species [18,31], tolerant macaques (*Macaca tonkeana* [16]) and rooks (*Corvus frugilegus* [32]). In canids, some studies have found evidence in wolves (both solicited and unsolicited [24,33]), whereas evidence in dogs remains inconsistent (evidence of solicited and unsolicited third-party affiliation in [28]; no evidence in [29]).

Several factors might affect the occurrence of PC behaviour. First, affiliative PC behaviour might be more likely to occur when the aggressor or third party have a closer bond to the former victim: for reconciliation, this might be especially important to repair and restore a valuable relationship, and reduce stress arising from uncertainty between closely bonded opponents; whereas for third-party affiliation, stronger bonds may increase the likelihood of receiving support, helping to reduce the risk of renewed aggression and lowering stress for both the victim and the third party. In dogs, for instance, reconciliation is more likely to occur between familiar than unfamiliar individuals [28]. In wolves, by contrast, there is no evidence that bond strength mediates the occurrence of reconciliation [25–27], although unsolicited third-party affiliation is more likely to occur between closely bonded individuals [33]. Secondly, the rank distance between former opponents may affect the probability of affiliative PC behaviour. In wolves, for example, one study found that reconciliation was more likely when the rank distance between opponents was greater, possibly because the negative consequences of a conflict might be stronger when the aggressor is relatively more dominant (e.g. in terms of renewed aggression [25]). However, other studies have not found this effect (e.g. in wolves [26,27]). Cordoni & Palagi [26], in particular, suggested that in species characterized by high levels of interdependence and cooperation, PC affiliative behaviour may occur largely independently of rank relationships, as maintaining group cohesion is critical for all individuals; by contrast, rank effects should be more pronounced in less tolerant, less cohesive social groups, where rank asymmetries and the associated costs of conflict may play a stronger role in shaping PC interactions. Finally, the likelihood of affiliative PC behaviour may depend on the characteristics of the conflict. In particular, when conflict intensity is high, and aggression is unidirectional, the negative consequences for the individuals involved and the group as a whole may be greater, making affiliative PC behaviours particularly important. In canids, for example, some studies reported that reconciliation is more likely following high-intensity conflicts (in one group of dogs [28]; in wolves [25]), although other studies found no such effect (in wolves [26,27]).

In this study, we investigated PC behaviour in wolves and wolves introgressed with dogs (i.e. second- or later-generation backcrosses of wolf-dog hybrids to wolves; see below). In wolves, pack members form highly cohesive social groups (e.g. [34–37]) and are highly dependent on each other, as they regularly cooperate to hunt, breed and defend their territory (e.g. [35,36,38–44]). Domestic dogs, by contrast, even when free-ranging, are facultatively social [45–47], as they are often solitary or form less cohesive groups and looser, more temporary relationships with each other [48–54] (but see [55]). Biologically speaking, wolves and dogs are the same species, and they can produce fertile offspring [56], therefore representing a case of introgressive hybridization. Through introgressive hybridization, some of the dogs’ social behavioural traits might disrupt the original wolves’ repertoire, increasing or decreasing the intensity and frequency of social interactions relative to the parental types [57]. In particular, if wolves inherit some of these social traits from dogs through introgression, admixed wolves may be expected to show looser social cohesion and lower interdependencies between group members, as recently indicated in captive groups [58].

Wolf-dog introgressive hybridization has been recognized as a serious threat to wolf conservation, particularly in Europe [59,60]. The introgression of genetic variants associated with domestication may not only compromise the genetic integrity of wolf populations and disrupt locally adaptive traits [61], but also subtly influence their behaviour and life history traits [62]. Although for practical conservation management wolf-dog hybrids are detected up to the second generation of backcross to wolves [63], this does not necessarily mean that further generations of backcrosses to wolves may not be affected by the introgression of domestic variants at both individual and social behaviour in nuanced yet fundamental ways. For example, hybridization and introgression from dogs may potentially alter pack cohesion and coordination, with potentially cascading effects for key ecological processes such as pup raising, hunting efficiency, territoriality and dispersal. Lobo and colleagues [64] identified fixed dog-derived introgression in Iberian wolves at the *MAST4* gene, which has been associated with several neurologic disorders, including developmental cognitive and motor delays, and may explain reduced dispersal capacity in this population. Establishing clear links between introgressed variants and phenotypic traits remains a major challenge in genomic research [64], particularly for behavioural traits, which are inherently difficult to observe and quantify in wild wolf populations.

In this context, we compared captive wolves and wolves introgressed with dogs to detect potential differences in their PC behaviour, which is a fundamental mechanism to maintain group cohesion. We formulated the following hypotheses and predictions. First, we hypothesized that affiliative PC behaviours are more likely in highly cooperative, stable groups, where mitigating the negative consequences of conflicts is particularly important to maintain cohesion and cooperation (H1) [6,24,25,27,65–67]. Accordingly, we predicted that both admixed and non-admixed wolves would engage in reconciliation and third-party affiliation (prediction 1a), but that these PC behaviours would be more likely to occur in non-admixed than in admixed wolves (prediction 1b). Second, we hypothesized that affiliative PC behaviours would be more likely when the negative consequences of aggression are greater, specifically when former opponents differ more strongly in rank (H2) [25]; when the relationship quality between the victim and the former aggressor/third party is particularly high (H3) [28,33] and when the preceding conflict is riskier, being unidirectional and more intense (H4) [25]. Therefore, we predicted that reconciliation and third-party affiliation would be more likely with higher rank distance between former opponents (prediction 2a), between dyads with stronger social bonds (prediction 3a), and following unidirectional, more intense conflicts (prediction 4). However, because wolves typically live in cohesive, highly interdependent groups, we also expected that the mediating role of social factors would differ between admixed and non-admixed wolves. In particular, we predicted that rank distance between former opponents would play a stronger mediating effect in admixed wolves (prediction 2b), and social bond strength would exert a stronger mediating effect in non-admixed wolves (prediction 3b). By comparing reconciliation and third-party affiliation in admixed and non-admixed wolves, this study may help clarify whether and how dog-introgression affects PC behaviour and the mechanisms that maintain group cohesion in wolves.

## 2. Materials and methods

### 2.1. Study subjects

We observed two groups of wolves (*n* = 20) and three groups of wolves introgressed with dogs (*n* = 13; hereafter, ‘admixed wolves’). Admixed individuals consistently displayed combinations of atypical morphological characteristics, including dark or pale coat colouration, depigmented nails and declaws [68–70], and originated from second- or later-generation backcrosses with wolves, as confirmed by genetic analyses based on biparental and/or uniparental markers [71]. During the study, all individuals had reached sexual maturity and were classified as adults (see table 1 for the composition of the study groups). All admixed wolves originated from wild-born individuals that had been removed as part of conservation management actions aimed at limiting hybridization in wild wolf populations [72], and were subsequently kept in captivity. All study subjects lived in relatively large enclosures together with conspecifics. In all groups, animals were regularly exposed to keepers and visitors and were typically fed every 1–3 days by leaving carcasses such as chickens, rabbits and other potential prey items in their enclosures.

**Table 1.** Location, sex, age (in years), rank and centrality of the captive admixed and non-admixed wolves included, and whether they were observed as aggressors and/or victims before the PC-MC sessions of this study. Rank and centrality values range from 0 to 1, reflecting the outcomes of dyadic agonistic interactions and an individual's integration within the social network (see text). A value of 0 corresponds to low-ranking, poorly integrated individuals, whereas a value of 1 corresponds to high-ranking, highly integrated individuals.

group	location	subject	sex	age	rank	aggressor or victim
non-admixed wolves	Kellerwald	amik	male	5	0.000	victim
		anouk	male	5	0.207	aggressor, victim
		bagira	male	4	0.188	victim
		cara	female	2	0.213	aggressor, victim
		charly	male	2	0.787	aggressor
		cowalski	male	2	0.515	aggressor
		noki	female	8	0.816	aggressor
		nuk	male	9	1.000	
	Osnabrück	angel	female	5	0.000	victim
		ano	male	5	0.033	victim
		eren	male	7	0.224	victim
		grey	female	4	0.015	aggressor, victim
		jackal	female	4	0.311	aggressor
		kaz	male	7	0.488	
		lagerta	male	5	0.549	aggressor
		noname	female	5	0.83	aggressor
		one	female	5	0.205	aggressor, victim
		ragnar	male	4	1.000	aggressor
		snow	male	7	0.123	aggressor, victim
		witcher	female	4	0.138	victim
dog-admixed wolves	Arcidosso	koda	female	≥8	0.204	victim
		luna	female	10	0.988	aggressor
		ora	female	≥8	1.000	aggressor
		rossa	female	10	0.000	victim
	Penne	baloo	male	7	0.048	aggressor, victim
		balto	male	7	0.657	aggressor
		licaone	male	7	0.000	victim
		lucio	male	7	0.454	aggressor, victim
		neo	male	7	1.000	aggressor
		syd	male	7	0.554	aggressor
	Semproniano	bo	male	11	0.481	victim
		nero	male	11	1.000	aggressor
		vincent	male	11	0.000	victim

The group at the WildtierPark Edersee consisted of eight European wolves (*Canis lupus lupus*), including a breeding pair and their offspring from three litters. Their 1.04 ha enclosure comprised grassland, shrubs and a forested hillside, where the visibility of the wolves was limited. All individuals in this group were intact during the study. The group at the Osnabrück Zoo consisted of 12 Hudson wolves (*Canis lupus hudsonicus*), including three males from a litter born at Amsterdam Zoo (The Netherlands) and nine individuals (six females and three males) from two litters born at the Osnabrück Zoo. Three of the six males had been vasectomized at the time of the study, leaving their testes and gonadal endocrine function intact. Their 0.45 ha enclosure comprised open woodland and a perimeter walkway, allowing relatively unobstructed observation of the group.

The group at the Parco Faunistico del Monte Amiata consisted of four wild-born female admixed wolves captured on separate occasions in Tuscany (G. Romeo 2026, personal communication), who were all introgressed at the nuclear loci. Two of the females were siblings originating from a wild admixed litter captured in 2014 near Siena, who were initially housed at the Centro di Recupero Animanatura Wild Sanctuary, from which they were transferred at the age of 8 years. A third sibling from the same litter died prior to the onset of this study. The remaining two females were captured as adults in 2012, one close to Firenze and one near Grosseto, and were initially housed in separate enclosures before being integrated with the other individuals in 2016 and 2022. All females were sterilized, with the two sisters having undergone ovariohysterectomy and the other two having undergone tubal ligation [58]. The enclosure at the Parco Faunistico del Monte Amiata covered around 9.00 ha and consisted of a grass clearing located on a hilltop surrounded by woodland. We conducted observations from a visitor turret, which allowed visibility over only a part of the enclosure. The group at the Centro di Recupero Animanatura Wild Sanctuary consisted of three wild-born male admixed wolves, all siblings from a litter of ten, who were captured in May 2014 close to Siena, when the pups were less than one week old [73]. They were all introgressed at nuclear loci and/or at the K-locus, and all shared a dog Y-haplotype. The litter was initially hand-raised and bottle-fed by caregivers for approximately one month, and then the males were transferred to the Centro di Recupero Animanatura Wild Sanctuary (initially with three of their sisters), where the males always remained together. All males were neutered via orchiectomy during their first year of life. Their enclosure covered approximately 0.30 ha and comprised a hill, a grass clearing, and a green wooded area beyond the hill that was not visible to the observer, who observed the group from a position in front of the clearing. The group at the Riserva Naturale Regionale Lago di Penne included six wild-born introgressed male siblings captured as pups at approximately one month of age in 2017 in the Gran Sasso–Monti della Laga National Park, originating from a dog–wolf admixed pack (F. Striglioni 2026, personal communication). All males were introgressed at nuclear loci and/or at the K-locus, and all shared a dog Y-haplotype. They were first housed in kennel boxes and then relocated at two and a half months of age to the enclosure at the Riserva Naturale Regionale Lago di Penne, which covered around 0.50 ha and included a grove and a pond. All individuals were vasectomized when they were six months of age and thus remained hormonally intact. The group was observed from an elevated visitor area that granted visual access to the majority of the enclosure.

### 2.2. Data collection

Behavioural observations were conducted across multiple sites and periods: at the WildtierPark Edersee in Kellerwald from January to May 2024, at the Osnabrück Zoo in Osnabrück from July to December 2023, at the Parco Faunistico del Monte Amiata in Arcidosso from September to October 2024, at the Centro di Recupero Animanatura Wild Sanctuary in Semproniano from May to September 2025, and at the Riserva Naturale Regionale Lago di Penne in Penne from April to August 2024. Data collection focused on three main aspects of behaviour. First, we assessed dyadic bond strength through 15-minute individual focal observations, based on the proportion of time spent in proximity to other group members. We observed each subject in 22 focal sessions, distributed across multiple days in a pseudorandomized order and at varying times of day, to reduce temporal biases in data collection. Second, we assessed group hierarchy and determined individual ranks using all-occurrence sampling. We recorded all visible instances of aggression (e.g. bites, chases and lunges), dominance behaviours (e.g. standing over a recumbent individual in a T-position, standing and placing a paw on it for >2 seconds) and submissive behaviours (e.g. licking a partner while showing a tucked tail and/or a hunched posture [43,74,75]), noting the date and time of the event, and the winner's and loser's identities.

Third, whenever we observed aggression in the group, we recorded the behaviour of the two opponents during a 2-minute PC period. We selected a 2-minute response window based on previous studies in wolves and dogs showing that PC behaviours typically occur within the first one to two minutes following the aggression [24,25,27,33]. Following Fraser *et al.* [14], if the same pair engaged in a new conflict during the PC, the observation was abandoned and restarted after the conflict ceased. To control for baseline affiliative behaviours, we conducted a 2-minute matched-control (MC) observation on the next possible day (and no later than 7 days after the PC), at the same time of day as the original PC. MCs were only conducted if no aggression had occurred in the preceding 15 minutes and if both former opponents were visible, awake and within approximately 10 meters of each other. If a suitable MC could not be identified within one week, the corresponding PC was removed from the dataset. To avoid circularity, aggressive encounters included in the PC-MC dataset were not used in analyses for establishing group hierarchy. In total, we collected 135 PC and 135 corresponding MC observations (admixed wolves: *n* = 59 + 59; non-admixed wolves: *n* = 76 + 76). Except for 18 of the 270 sessions, which were coded live, all observations were video-recorded and subsequently coded for analyses using the videos.

### 2.3. Data coding

For each PC-MC pair, we first recorded the characteristics of the aggression, including: (i) the identity of the victim, (ii) the identity of the aggressor, (iii) whether the aggression was unidirectional (i.e. directed solely from the aggressor to the victim) or bidirectional (i.e. the victim also displayed aggressive behaviour), and (iv) the intensity of the aggression, classified as high (i.e. involving physical contact) or low (i.e. without physical contact; figure 1). We further recorded PC affiliative behaviour involving the former victim, including: (v) all affiliative behaviours received by the victim within the 2 minutes following the aggression, (vi) the identity of the partners involved, (vii) the type of affiliative behaviour (i.e. body contact, social play, lick and sniff [24,26]; figure 1), (viii) whether the victim solicited the interaction, and (ix) the latency between the end of the aggression and the occurrence of each affiliative behaviour. Affiliative behaviours directed by the former aggressor towards the victim were considered potential instances of reconciliation, whereas affiliative behaviours directed by third parties towards the victim were considered potential instances of third-party affiliation.

### 2.4. Statistical analyses

We used R (version 4.4.2) [76] to conduct all the analyses. First, for each possible pair of individuals, we calculated dyadic bond strength using the Composite Sociality Index (CSI) [4]. To this aim, we divided the proportion of time two individuals spent in proximity by the mean proportion of time all dyads in the group spent in proximity. CSI below 1 indicated weaker-than-average relationships, and CSI above 1 indicated stronger-than-average relationships. Second, we used the Elo-rating method (EloRating package, version 0.43) to assess the dominance hierarchy of each study group and the ranks of all group members (table 1). We calculated Elo scores based on all dyadic agonistic interactions with a clear winner-loser outcome that were not included in the PC-MC datasets. We then standardized Elo scores to vary from 0 (i.e. low-ranking individuals) to 1 (i.e. high-ranking individuals).

Third, to assess whether both admixed and non-admixed wolves engage in reconciliation and third-party affiliation (prediction 1a), we calculated corrected conciliatory tendencies (CCTs). We constructed a first dataset by extracting, for each PC-MC pair, the latency of the first affiliative interaction between former opponents in PC and MC, as well as the latency of the first affiliative interaction between a third party and the former victim in PC and MC [77]. We then compared the latencies in PC and MC and categorized each PC-MC pair as: (i) attracted, if the affiliation occurred only in PC or earlier in PC than in MC; (ii) dispersed, if the affiliation occurred only in MC or earlier in MC than in PC; or (iii) neutral in all other cases. For each individual who was the victim of aggression, we then calculated two measures of CCTs: one for affiliative interactions involving the former aggressor (reconciliation), and one for affiliative interactions involving a third party (third-party affiliation). Individual CCTs were calculated by subtracting the number of dispersed PC-MC pairs from the number of attracted PC-MC pairs, and dividing the result by the sum of attracted, dispersed and neutral PC-MC pairs for that individual [78]. We then used non-parametric Mann–Whitney *U*-tests to compare CCTs for reconciliation and third-party affiliation between admixed and non-admixed wolves [79]. All tests were two-tailed with the α level set at 0.05.

Finally, we used the glmmTMB package [80] to run two sets of generalized linear mixed models (GLMMs). For the first set of models, the dataset included one line for each PC-MC pair that was either attracted or dispersed (*n* = 111). For each line, we entered the identities of the victim and the individual first providing affiliative behaviour in the PC-MC pair, introgression (i.e. admixed versus non-admixed wolf), whether the first affiliative behaviour was provided by the former aggressor (reconciliation) or by a third party (third-party affiliation), whether affiliation occurred only/earlier in PC than in MC, or not (coded 1/0), the rank distance between former opponents, and the social bond strength between the victim and the individual providing affiliative behaviour. We then ran a full model with a binomial error distribution to model the log-odds of affiliative behaviour occurring only/earlier in PC. As test predictors, we included two different three-way interactions (along with all corresponding two-way interactions and main effects): the interaction between introgression, affiliation type (i.e. reconciliation and third-party affiliation) and rank distance between former opponents, and the interaction between introgression, affiliation type and social-bond strength between the victim and the individual providing affiliative behaviour. As random intercepts, we included the identity of the victim and of the individual providing affiliative behaviour. We did not include group identity as a random intercept, as the number of groups was too small (*n* = 5) to reliably estimate variance components. This model allowed testing whether affiliative PC behaviour was more likely in non-admixed than in admixed wolves (prediction 1b: introgression effect), when rank distance between former opponents was higher (prediction 2a: rank distance effect), and when the victim had a closer bond to the individual providing affiliation (Prediction 3a: social bond strength effect). Moreover, it allowed testing whether the effect of rank distance was stronger in admixed than non-admixed wolves (prediction 2b: effect of the two-way interaction between introgression and rank distance), and whether the effect of social bond strength was stronger in non-admixed than admixed wolves (prediction 3b: effect of the two-way interaction between introgression and social bond strength). The inclusion of affiliation type in the three-way interactions further allowed testing all these predictions separately for reconciliation and third-party affiliation.

For the second set of models, the dataset included one line for each PC-MC pair, including attracted, dispersed and neutral pairs (*n* = 135). In the dataset, we specified the aggressor and victim identities, introgression (i.e. admixed versus non-admixed wolf), whether the first affiliative behaviour (from the former aggressor or a third party) occurred only in PC or earlier in PC than in MC (coded 1/0), and the directionality and intensity of the aggression. We then ran a full model with a binomial error distribution to model the log-odds of affiliative behaviour occurring only/earlier in PC. As test predictors, we included the two-way interactions of introgression with aggression directionality and aggression intensity. As random intercepts, we included victim and aggressor identities. This model allowed us to test whether, in both admixed and non-admixed wolves, affiliative PC behaviours are more likely following unidirectional, more intense conflicts (prediction 4).

Each full model was compared with a corresponding null model, which included only random intercepts and control variables but no test predictors, using likelihood ratio tests [81]. We assessed the contribution of individual predictors using the drop1 function. Non-significant interactions were removed, and models were refitted retaining only lower-order (nested) interactions and main effects. For significant categorical variables, we conducted post hoc pairwise comparisons using the emmeans package [82]. Model diagnostics were performed with the DHARMa [83] and performance [84] packages. The models included in this study had no problems regarding residual distribution, convergence, overdispersion or multi-collinearity (maximum VIF across models = 1.18; [85]). More complex models could not be fitted owing to convergence issues.

## 3. Results

### 3.1. Characteristics of the aggression

We recorded 135 conflicts, 59 in admixed wolves and 76 in non-admixed wolves. No injuries resulted from these conflicts. These conflicts involved 20 different aggressors (eight admixed wolves, 12 non-admixed wolves) and 18 different victims (seven admixed wolves, 11 non-admixed wolves). Most conflicts were unidirectional (admixed wolves: 86% or 51/59; non-admixed wolves: 75% or 57/76) and involved physical contact (admixed wolves: 64% or 38/59; non-admixed wolves: 62% or 47/76).

### 3.2. Characteristics of the affiliative behaviour exchanged after conflicts

Most PC affiliative behaviour consisted of licks, sniffs and body contact. Both admixed and non-admixed wolves showed licks and sniffs, whereas body contact was observed only in admixed wolves. In particular, the occurrence of licks in PC was relatively higher compared to MC both in admixed wolves (79% versus 21% or 84/22 licks in PC/MC) and non-admixed wolves (85% versus 15% or 46/8 licks in PC/MC). Similarly, the occurrence of sniffs in PC was relatively higher compared to MC in admixed wolves (74% versus 26%, or 31/11 sniffs in PC/MC) and non-admixed wolves (67% versus 33% or 6/3 sniffs in PC/MC). In non-admixed wolves, the occurrence of body contact in PC was also relatively higher compared to MC (74% versus 26% or 17/6 body contacts in PC/MC). On average (mean ± s.d.), the first affiliative behaviour in a session occurred during PC, with a latency of 21 ± 28 seconds in admixed wolves and 12 ± 19 seconds in non-admixed wolves. In MC, the latency was 25 ± 26 seconds in admixed wolves and 41 ± 19 seconds in non-admixed wolves. The temporal distribution of the first affiliative behaviour in PC and MC is depicted in figure 2.

**Figure 1.**
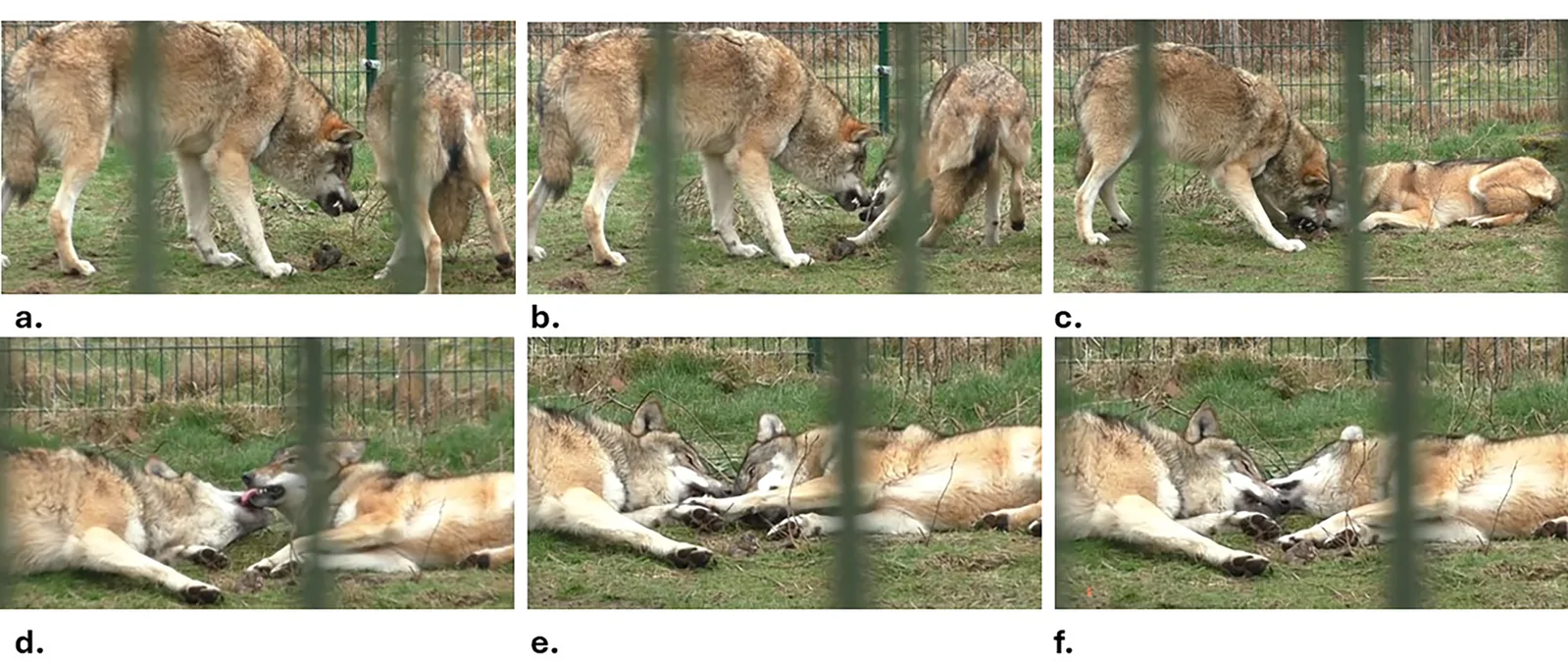
Video frames of two non-admixed wolves from the Kellerwald group, Charly (left) and Cara (right), showing (a–c) aggressive behaviour and (d–f) affiliative PC behaviour. (a–b) Low-intensity unidirectional aggression (i.e. directed solely from Charly towards Cara, without physical contact); (c) high-intensity unidirectional aggression (i.e. directed solely from Charly towards Cara, involving physical contact); (d) licking, (e) body contact, and (f) sniffing, directed from the former victim Cara towards the former aggressor Charly.

**Figure 2.**
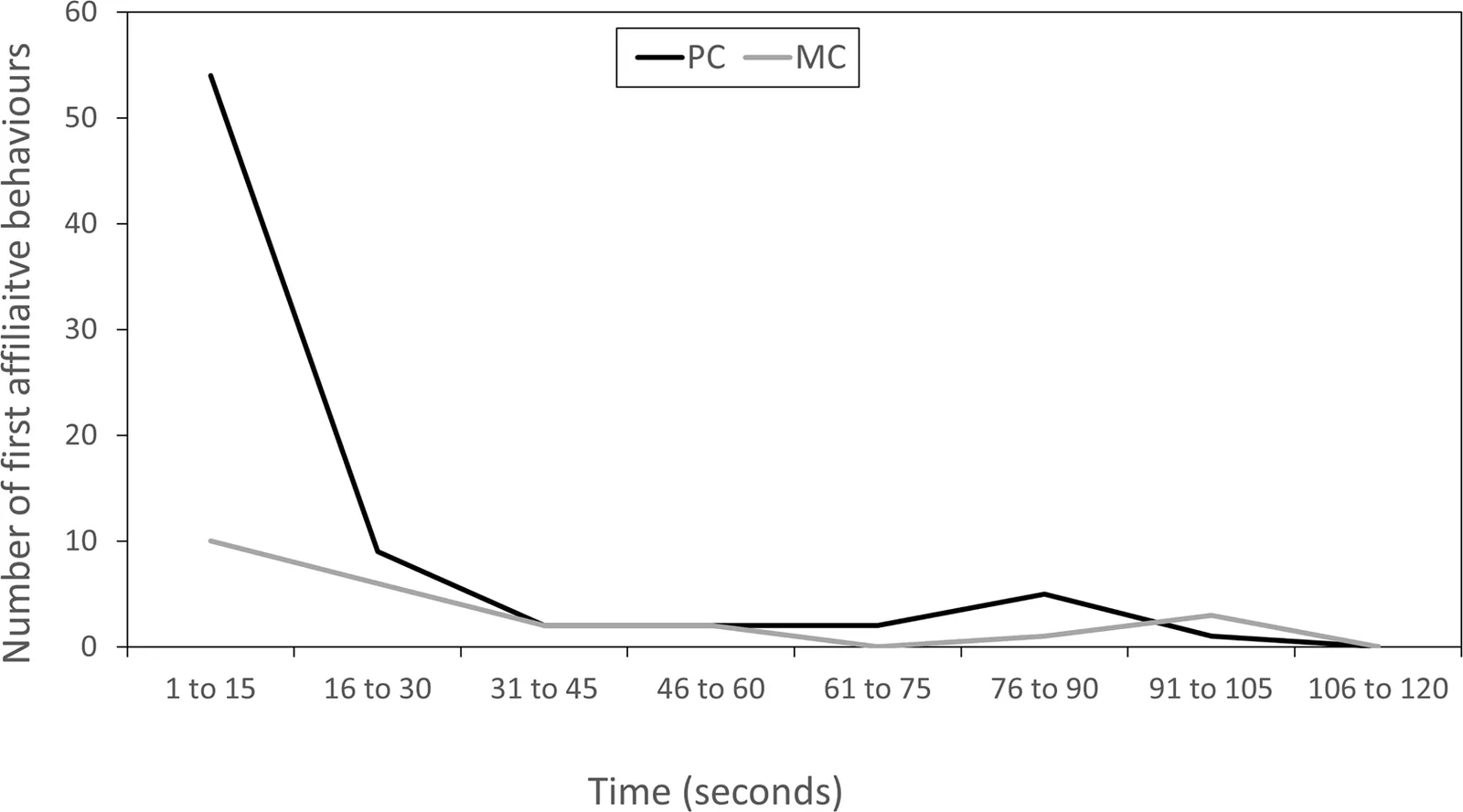
Temporal distribution of the first affiliative behaviour involving the former victim of an aggression in both post-conflict (PC) and matched-control (MC) sessions, for all the individuals and groups observed.

### 3.3. Occurrence of reconciliation and third-party affiliation

Out of all the conflicts observed, 29% (39/135) were first followed by reconciliation (i.e. the first affiliative behaviour was produced only in PC or earlier in PC than in MC, and involved the former opponents). Moreover, 23% (31/135) of the conflicts were first followed by third-party affiliation (i.e. the first affiliative behaviour was produced only in PC, or earlier in PC than in MC, and involved the victim and a third party). Finally, 48% (65/135) were not resolved (i.e. affiliative behaviour towards the victim was not produced in PC, or it was produced earlier or at the same time in MC as in PC). In particular, admixed wolves showed reconciliation first after 36% (21/59) of the conflicts and third-party affiliation first after 22% (13/59) of the conflicts, whereas non-admixed wolves showed reconciliation first after 22% (18/76) of the conflicts and third-party affiliation first after 22% (18/76) of the conflicts. In some cases, both reconciliation and third-party affiliation occurred. These sequences were relatively more frequent in admixed wolves (12% or 7/59 conflicts included first a reconciliation and then a third-party affiliation, while 10% or 6/59 conflicts included first a third-party affiliation and then a reconciliation) than in non-admixed wolves (4% or 3/76 and 5% or 4/76, respectively). Both in admixed and non-admixed wolves, the majority of reconciliation and third-party affiliation events were solicited by the former victim. In admixed wolves, victims solicited reconciliation in 90% (19/21) of the events and third-party affiliation in 77% (10/13) of the events, whereas in non-admixed wolves, victims solicited reconciliation in 88% (15/17) of the events and third-party affiliation in 89% (16/18) of the events.

### 3.4. Corrected conciliatory tendencies

Out of the 18 different victims observed during the study (seven admixed wolves, 11 non-admixed wolves), 89% (16/18) showed positive CCTs for both reconciliation and third-party affiliation. Only two individuals showed negative CCTs for reconciliation (Bo, one admixed wolf, and Amik, one non-admixed wolf), and two for third-party affiliation (Amik and Bagira, both wolves). On average, CCT for reconciliation was 12% in admixed wolves and 29% in non-admixed wolves, whereas CCT for third-party affiliation was 9% in admixed wolves and 25% in non-admixed wolves. When comparing individual CCTs for reconciliation between admixed and non-admixed wolves, we found no significant differences (Mann–Whitney *U*-test: *W* = 31.5, *n*₁ = 7, *n*₂ = 11, *p* = 0.549). The same was true for third-party affiliation (Mann–Whitney *U*-test: *W* = 30.5, *n*₁ = 7, *n*₂ = 11, *p* = 0.477).

### 3.5. Social factors affecting the log-odds of PC affiliative behaviour

The first full model significantly differed from the corresponding null model (GLMM, *χ*^2^ = 16.55, d.f. = 6, *p* = 0.011; table 2). In particular, the log-odds of affiliative behaviour occurring only/earlier in PC than in MC were significantly affected by the two-way interactions of introgression with rank distance (*p* = 0.031) and with social bond strength (*p* = 0.010). As affiliation type had no significant mediating effect on these interactions, these patterns were similar for both reconciliation and third-party affiliation, although affiliation type showed a marginal main effect (*p* = 0.055), suggesting that the log odds of reconciliation were tendentially higher than those of third-party affiliation. The two-way interactions showed that the effect of rank distance on the log-odds of reconciliation or third-party affiliation differed between admixed and non-admixed wolves: while PC affiliative behaviour was more likely to occur with increasing rank distance in admixed wolves, this effect was absent in non-admixed wolves (figure 3). Moreover, the effect of social bond strength on the log-odds of reconciliation or third-party affiliation differed between admixed and non-admixed wolves: while PC affiliative behaviour was more likely to occur with increasing bond strength in non-admixed wolves, this effect was absent in admixed wolves (figure 4), suggesting that stronger social bonds promoted affiliative PC interactions in non-admixed wolves but not in admixed wolves.

**Table 2.** For each test predictor and control (in italics), estimates, standard deviation (s.d.), 95% confidence intervals (CIs), LRT, d.f. and *p*-values of our two full models, with asterisks denoting significant test predictors. We do not report LRT, d.f. and *p-*values for the intercepts and for all the main terms that were also involved in an interaction.

predictors	estimate	SE	95% CIs	LRT	d.f.	*P*
**model 1: log-odds of affiliative behaviour occurring only/earlier in PC than in MC, for all attracted and dispersedPC-MC pairs (GLMM, *χ*^2^ = 16.55, d.f. = 6, *p* = 0.011)**						
intercept	0.76	0.42	–0.06 to 1.58	—	—	—
affiliation type (reconciliation)	1.06	0.57	–0.05 to 2.18	3.67	1	0.055
introgression (non-admixed wolf)	0.83	0.63	–0.41 to 2.06	—	—	—
rank distance	6.16	2.74	0.78 to 11.53	—	—	—
bond strength	–0.98	0.75	–2.45 to 0.50	—	—	—
introgression (non-admixed wolf) × rank distance	–7.24	3.53	–14.15 to –0.32	4.67	1	0.031*
introgression (non-admixed wolf) × bond strength	3.29	1.21	0.91 to 5.67	6.58	1	0.010*
**model 2: log-odds of affiliative behaviour occurring only/earlier in PC than in MC, for each PC-MC pair (GLMM, *χ*^2^ = 11.89, d.f. = 4, *p* = 0.018)**						
intercept	–0.16	0.77	–1.67 to 1.35	—	—	—
introgression (non-admixed wolf)	0.26	0.96	–1.63 to 2.14	—	—	—
aggression directionality	2.54	1.47	–0.34 to 5.42	—	—	—
aggression intensity	1.14	0.54	0.09 to 2.20	4.47	1	0.034*
introgression (non-admixed wolf) × aggression directionality	–4.15	1.70	–7.47 to –0.82	8.81	1	0.003*

**Figure 3.**
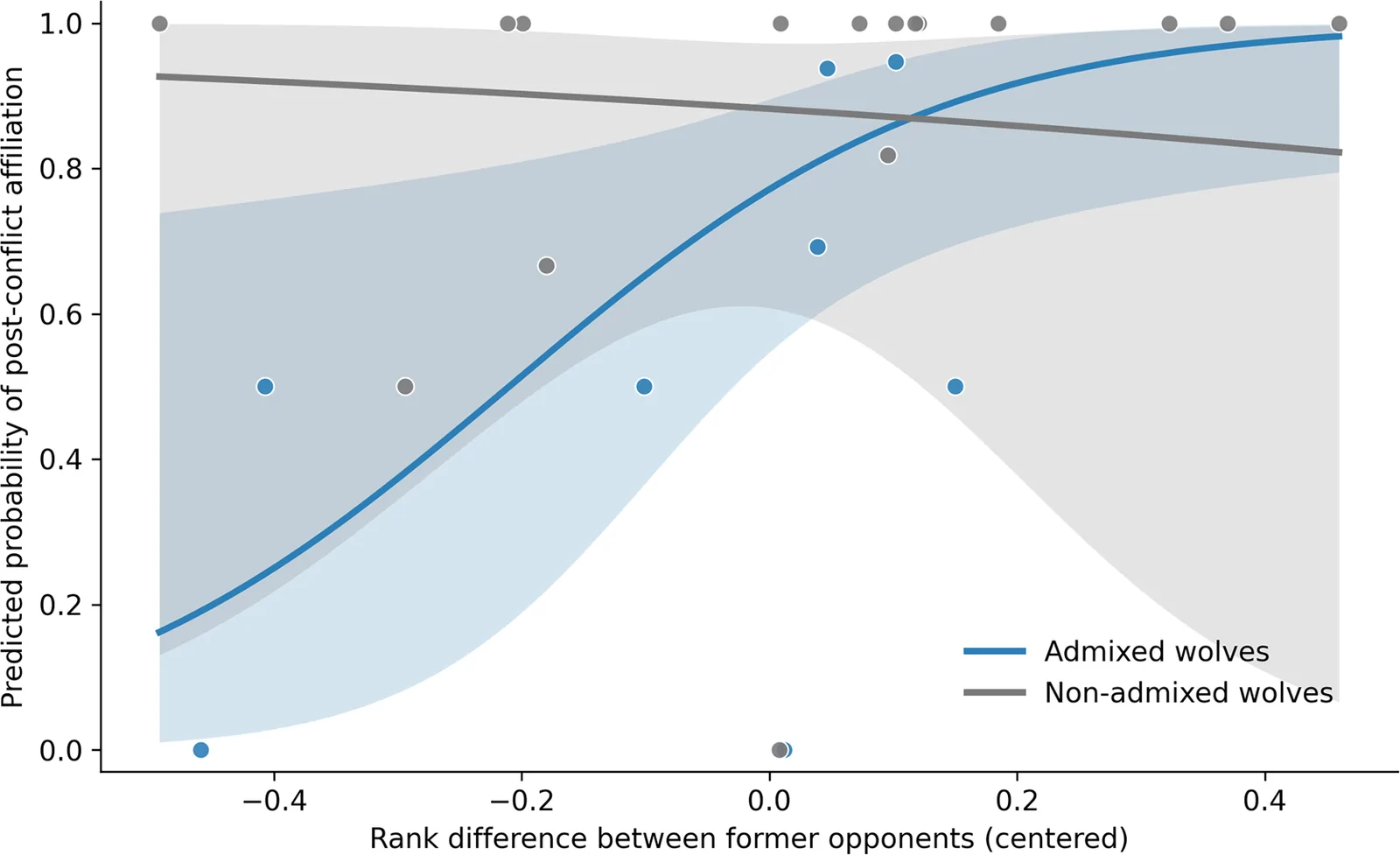
For admixed (in blue) and non-admixed wolves (in grey), predicted probability of reconciliation or third-party affiliation as a function of rank distance between former opponents. The lines represent the fitted model, as described in the text, and the shaded areas the 95% confidence intervals. Dots represent the mean probability of showing PC affiliation for each observed dyad, with data points aggregated within dyads.

**Figure 4.**
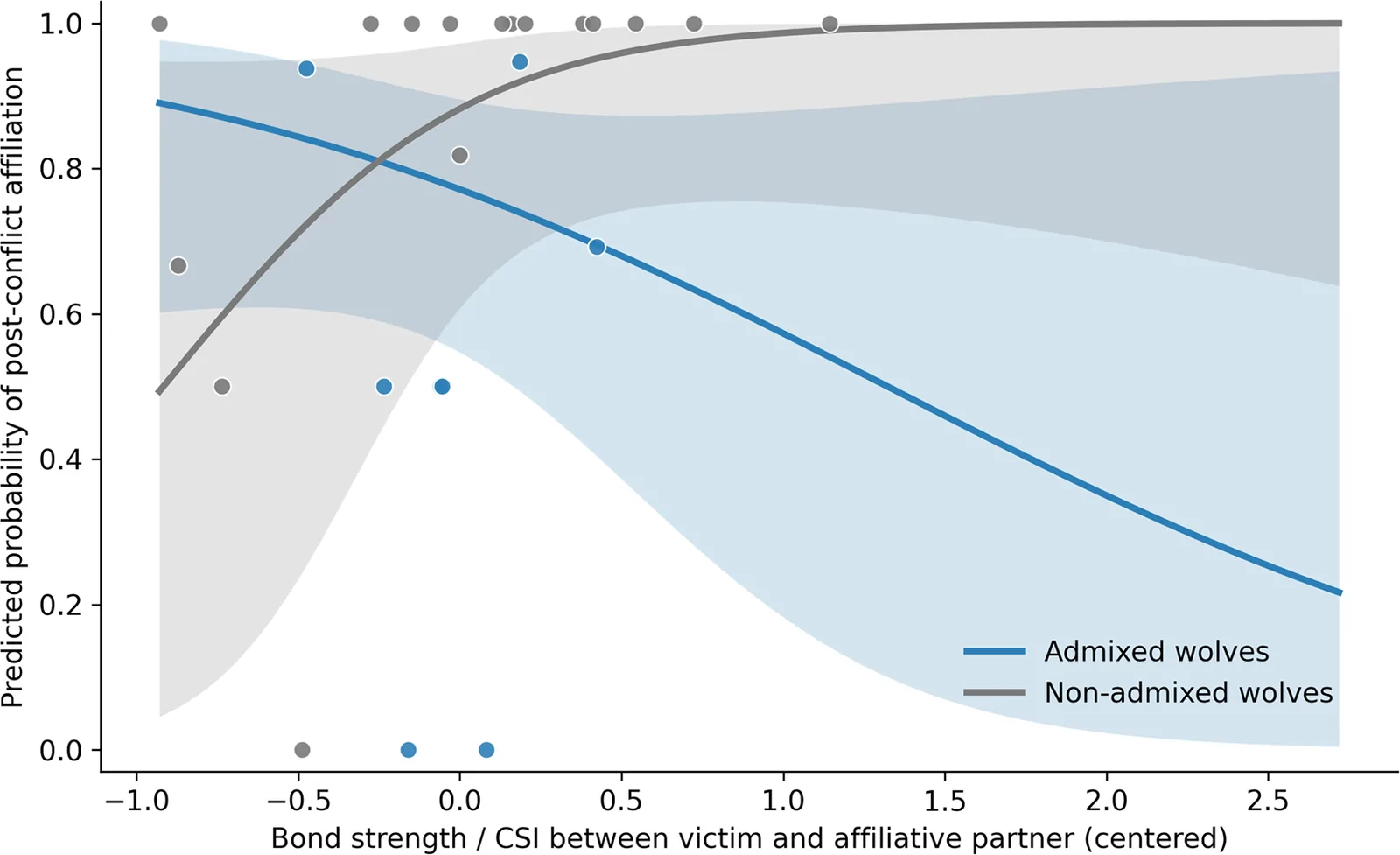
For admixed (in blue) and non-admixed wolves (in grey), predicted probability of reconciliation or third-party affiliation as a function of social bond strength between the former victim and the individual involved in affiliative behaviour. The lines represent the fitted model, as described in the text, and the shaded areas the 95% confidence intervals. Dots represent the mean probability of showing PC affiliation for each observed dyad, with data points aggregated within dyads.

### 3.6. Characteristics of the aggression affecting the log-odds of PC affiliative behaviour

The second full model significantly differed from the corresponding null one (GLMM, *χ*^2^ = 11.89, d.f. = 4, *p* = 0.018). In particular, the log-odds of affiliative behaviour occurring only/earlier in PC than in MC were significantly affected by the two-way interaction of introgression with aggression directionality (*p* = 0.003), and by aggression intensity (*p* = 0.034). In particular, post hoc tests showed that the log-odds of reconciliation or third-party affiliation were significantly higher after unidirectional than bidirectional conflicts in non-admixed wolves (*p* = 0.035), but not in admixed wolves (*p* = 0.084; figure 5). Finally, PC affiliative behaviour was more likely to occur after high-intensity conflicts, both in admixed and non-admixed wolves (figure 6).

**Figure 5.**
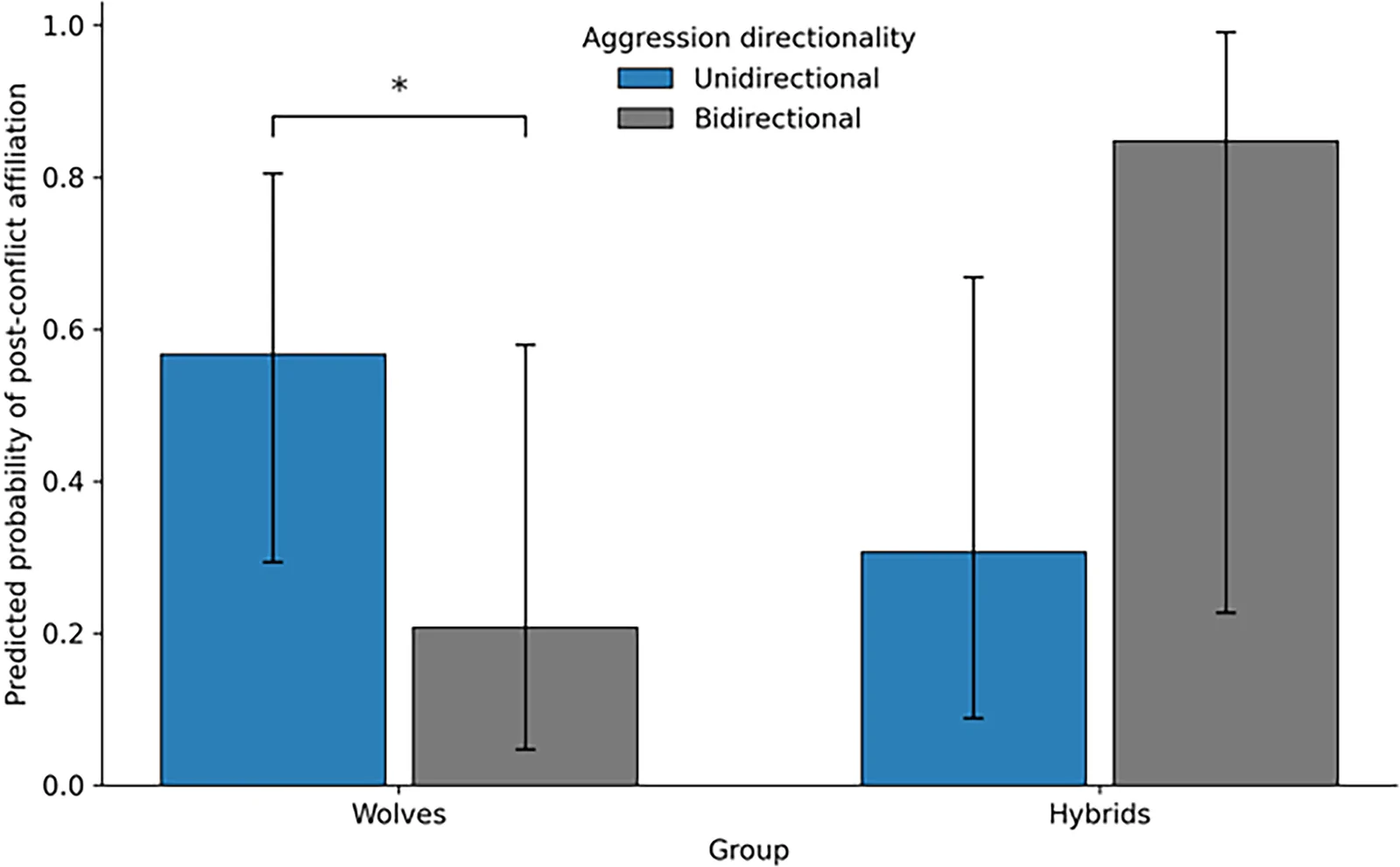
For admixed and non-admixed wolves, predicted probability of reconciliation or third-party affiliation when aggression was unidirectional (in blue) or bidirectional (in grey). Bar heights represent model-estimated mean probabilities, and error bars indicate 95% confidence intervals. Post hoc significant differences are marked with an asterisk.

**Figure 6.**
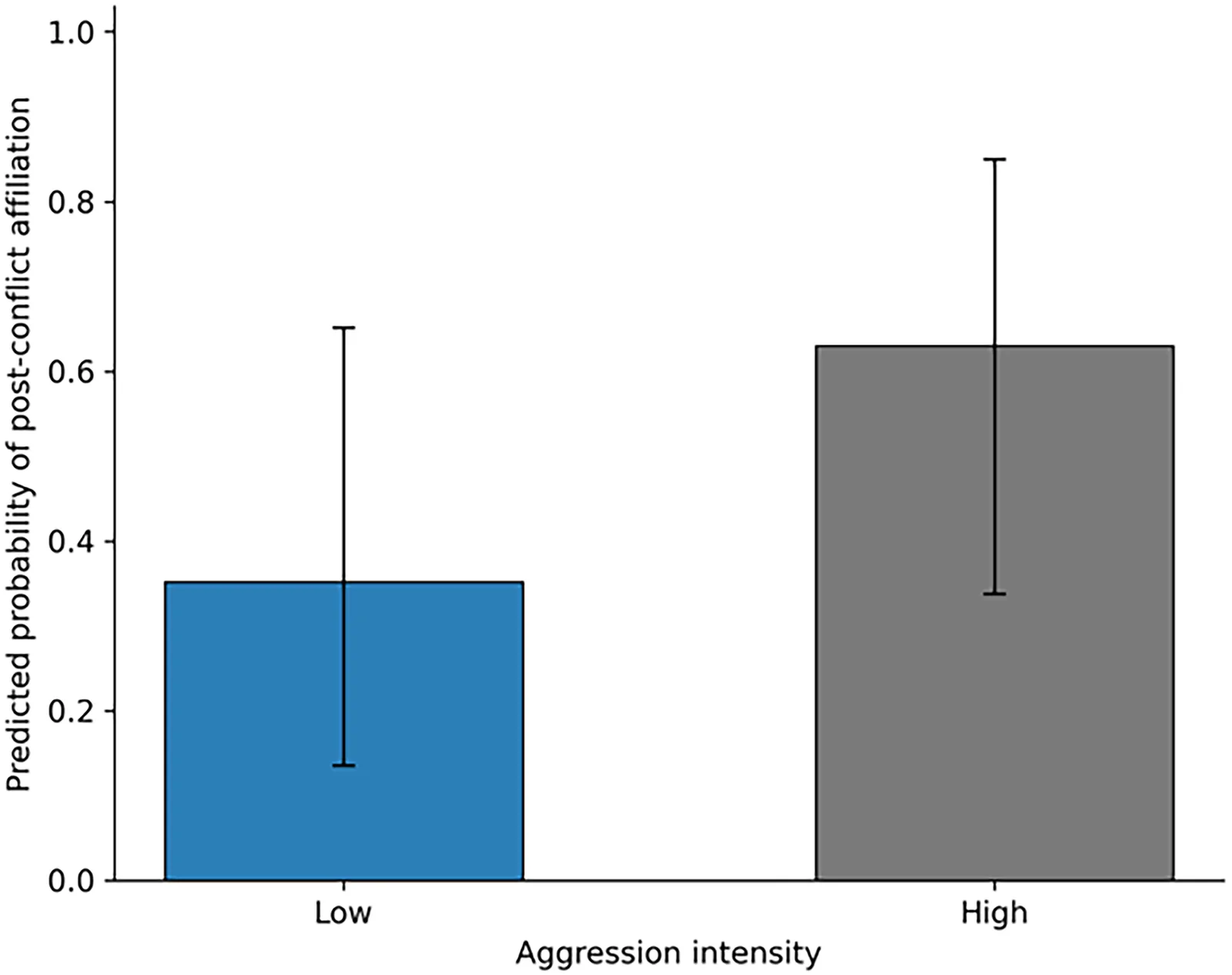
For all study individuals, predicted probability of reconciliation or third-party affiliation when the intensity of the aggression was low (in blue) or high (in grey). Bar heights represent model-estimated mean probabilities, and error bars indicate 95% confidence intervals.

## 4. Discussion

Our study provided evidence of PC affiliation consistent with reconciliation and third-party affiliation in both admixed and non-admixed wolves. Most affiliative interactions occurred shortly after conflicts, often within the first minute, and were typically initiated by the former victim. The majority of individuals had positive CCTs for both reconciliation and third-party affiliation, and although average CCTs were higher in non-admixed than in admixed wolves, this was not significantly so. The probability of PC affiliation depended on both social factors and characteristics of the aggression, with differences between admixed and non-admixed wolves. In non-admixed wolves, PC affiliation was more strongly associated with the quality of social relationships, whereas rank asymmetries appeared to play a more prominent role in admixed wolves. Moreover, PC affiliation was more likely following high-intensity aggression in both admixed and non-admixed wolves, and after unidirectional aggression, but only in the latter. Overall, these results indicate that while both admixed and non-admixed wolves engage in PC affiliation in captivity, the specific mechanisms underlying these behaviours partially differ between the two, pointing to differences in how social factors and conflict characteristics regulate PC behaviour.

In line with our first prediction that affiliative PC behaviours are present in social groups (prediction 1a), we found evidence that both admixed and non-admixed wolves show reconciliation and third-party affiliation. First, affiliative behaviours occurred with shorter latencies after a conflict (PC) than in MC sessions, in non-admixed and partially in admixed wolves. Second, around half of the conflicts were followed by reconciliation or third-party affiliation, both in admixed and non-admixed wolves. Third, individual CCTs were positive for 89% of the victims observed, both for reconciliation and third-party affiliation, suggesting that the largest majority of individuals showed a tendency to engage in PC affiliative behaviour. Our findings are in line with previous studies in wolves providing evidence of reconciliation [24–27] and third-party affiliation [24,33]. Moreover, our results show that affiliative PC behaviours are not disrupted by the introgression of dog genes, although evidence of reconciliation and third-party affiliation is more controversial in dogs (positive evidence for reconciliation: [28,29]; for third-party affiliation: [28]; negative evidence for reconciliation: [25]; for third-party affiliation: [29]). The occurrence of affiliative PC behaviours in both admixed and non-admixed wolves is not surprising, as these behaviours appear essential to mitigate the negative consequences of conflicts and may thus be widespread in social species (e.g. [6,24,27,67]).

In both admixed and non-admixed wolves, the majority of reconciliation and third-party affiliation events were solicited by the former victim. For reconciliation, this pattern is consistent with previous findings in wolves, where reconciliation is typically initiated by the victim [24,25,27]. For third-party affiliation, the fact that most interactions were initiated by the victim (77% of conflicts in admixed wolves and 89% in non-admixed wolves) shows that third parties do not typically initiate interactions with former victims spontaneously, and that it is instead the victims who actively seek affiliation, leaving little room for the hypothesis that these interactions may represent real consolation (see [16] for a discussion). Overall, it appears that, in admixed and non-admixed wolves, victims are likely to seek affiliative interactions with other group members after a conflict, whether with former opponents or with third parties. This pattern may also help explain why, in our study, reconciliation was slightly more frequent in admixed (36%) than in non-admixed wolves (22%), as the smaller group sizes in admixed wolves might have limited the availability of alternative partners, increasing the likelihood that victims re-engaged with the former aggressor rather than other group members. Although most instances of PC affiliative behaviour in our study were solicited, we also observed several unsolicited interactions, in line with previous studies in wolves reporting both solicited and unsolicited PC affiliation [24,26,33]. Future studies including a larger number of both solicited and unsolicited interactions would be valuable to better assess potential functional differences between these behaviours [33].

Our study provided no support for the prediction that affiliative PC behaviours would be more likely in non-admixed wolves than in admixed wolves (prediction 1b). Although, on average, CCTs were more than twice as high in non-admixed wolves as in admixed wolves for both reconciliation and third-party affiliation, this difference did not reach statistical significance. The fact that CCTs were higher in non-admixed than in admixed wolves, despite the difference not being statistically significant, is not in contrast with the observation that a higher percentage of conflicts were followed by reconciliation in admixed wolves: indeed, admixed wolves had a higher proportion of attracted pairs, but an even higher proportion of dispersed pairs compared to non-admixed wolves. By accounting for dispersed pairs, CCTs provide a more accurate measure of PC affiliative tendencies, as they are independent of the baseline level of interactions [78]. In our study, mean CCTs were 25–29% in non-admixed wolves (for third-party affiliation and reconciliation) and 9–12% in admixed wolves. These values are lower than those previously reported in two studies on reconciliation in wolves (47% in [27]; 53% in [26]), but in the range usually reported in other studies (e.g. for reconciliation, 22% in wolves [25]; 17% in hyenas [22]; 16% in chimpanzees [86]; 31% in common marmosets [87]; for third-party affiliation, 29% in wolves [33]). Rather than indicating a general reduction of PC affiliation in admixed wolves, our findings suggest that admixed and non-admixed wolves may differ in the social and conflict-related factors associated with the occurrence of PC affiliation. Therefore, in the future, it will be important to further explore inter-group variation in PC behaviour, including the socio-ecological drivers of such variation. In our study groups, we observed partial differences in the types of affiliative behaviours used after conflicts: whereas body contact was only observed in non-admixed wolves, both admixed and non-admixed wolves exhibited sniffing and licking (as also reported for wolves in [24]). In the future, it would be interesting to further investigate whether the lack of body contact as an affiliative PC behaviour in admixed wolves generalizes to other groups, and whether such differences reflect broader variations in social strategies or bonding patterns between admixed and non-admixed wolves.

Our findings support the predictions that affiliative PC behaviour increases with greater rank distance between former opponents (prediction 2a) and with stronger social bonds (prediction 3a), as these might increase the risk of renewed aggression and the potential stress and damage caused by the conflict [25,28,33]. We also found support for the prediction that these effects differ between admixed and non-admixed wolves, with rank distance promoting PC affiliation in admixed wolves (prediction 2b), and stronger social bonds promoting affiliation in non-admixed wolves (prediction 3b). In line with [26] and with similar findings in spotted hyenas [22], which are also highly cooperative and form structured, cohesive groups [88,89], non-admixed wolves in our study showed affiliative PC behaviour independently of rank distance. Cordoni & Palagi [26] suggested that in highly interdependent and cooperative groups, conflict resolution and the maintenance of group cohesion are critical for all individuals, reducing the influence of rank asymmetries. By contrast, in less cohesive groups, rank differences may more strongly shape the costs of conflict and, consequently, the likelihood of PC affiliation [26]. This interpretation is consistent with evidence from the wild that agonistic interactions in wolves rarely escalate [43], suggesting the presence of effective conflict-management mechanisms. The finding that affiliative PC behaviour in non-admixed wolves was more likely in dyads with stronger social bonds is consistent with studies in other species showing that individuals are more likely to reconcile when their relationship is valuable [90,91], and with evidence in wolves that unsolicited third-party affiliation occurs more frequently between closely bonded individuals [33]. Overall, these findings suggest that, in non-admixed wolves, PC affiliation may be driven more by the need to repair valuable social relationships and mitigate the higher stress associated with conflicts between closely bonded individuals than by the risk of renewed aggression. Finally, the fact that these patterns did not differ between interactions involving former opponents and those involving third parties suggests that the mechanisms underlying reconciliation and third-party affiliation may be largely similar for this species.

Largely in line with our predictions, the characteristics of the conflict influenced the likelihood of PC affiliation (prediction 4). In both admixed and non-admixed wolves, reconciliation and third-party affiliation were more likely after more intense conflicts, which may reflect the higher risks associated with such interactions, including an increased likelihood of injury, higher stress levels and greater uncertainty about the stability of the relationship. Our findings are in line with previous studies showing higher rates of reconciliation following high-intensity conflicts in both wolves [25] and dogs [28]. Only partially in line with our predictions, we also found an effect of aggression directionality, but only in non-admixed wolves: unidirectional conflicts, which are typically associated with greater asymmetry and potential risk for the victim, were more likely to be followed by PC affiliation, whereas no differences were detected for admixed wolves.

Our study has several limitations. First, our data were collected in captivity. Although such a study would not currently be feasible in the wild owing to the lack of observable groups with admixed wolves, caution is warranted when generalizing these findings to wild populations, where socio-ecological conditions differ substantially. Second, the number of study groups and individuals was necessarily limited, reflecting the rarity of facilities housing admixed wolves. As a result, variation in group size, composition, enclosure conditions and early life experiences could not be controlled for, and their contribution to the observed variation cannot be excluded. Groups of non-admixed wolves, in particular, were slightly larger than those of admixed wolves, raising the possibility that our findings also reflect group size effects, as mechanisms promoting conflict resolution, such as reconciliation and third-party affiliation, may be especially important in larger groups to maintain cohesion. Future studies including a greater number of groups and a wider range of group sizes are therefore needed to determine whether our findings reflect introgression or rather the specific socio-ecological conditions of the study groups. Finally, along the same lines, some captive facilities might house admixed and non-admixed wolves together, or individuals with substantially different levels of dog introgression. Assessing such introgression and including such groups in future studies could provide further insight into how aspects of social behaviour may be affected by the specific group composition and degree of admixture.

In conclusion, our findings provide preliminary evidence that, while both mixed and non-admixed wolves engage in PC affiliation in captivity, the mechanisms underlying these behaviours may partially differ: while stronger social bonds promoted affiliation in non-admixed wolves, greater rank distance was associated with higher PC affiliation in admixed wolves. While these effects appear subtle, they suggest potential changes in the mechanisms underlying conflict management and social cohesion. From a conservation perspective, such differences could have important implications for wolf groups living in the wild, as dog introgression might partially alter mechanisms that are essential to maintain group cohesion in the face of conflict [6,7]. Changes in these behavioural patterns could, in turn, influence key ecological processes such as pack coordination and cooperation during hunting, breeding and territorial defence, which are vital for wolves (e.g. [35,36,38,40–44]). Given the potential threat that introgression with dogs poses to European wolf populations [62], further studies examining how introgression may affect wolf social behaviour are needed, ideally in natural settings. In particular, future research combining behavioural, ecological and genomic approaches will be crucial to assess the genomic mechanisms underlying behavioural variation and understand whether such variation scales up to significantly affect group cohesion and cooperation in wolf populations. Such insights will be essential to inform effective and ethically sound wolf-conservation strategies.

## Supplementary Material



## Data Availability

Data and code are available as supplementary material [92].
